# Differential Cytokine Levels during Normothermic Kidney Perfusion with Whole Blood- or Red Blood Cell-Based Perfusates—Results of a Scoping Review and Experimental Study

**DOI:** 10.3390/jcm11226618

**Published:** 2022-11-08

**Authors:** Julie De Beule, Delphine Keppens, Hannelie Korf, Ina Jochmans

**Affiliations:** 1 Laboratory of Abdominal Transplantation, Transplantation Research Group, Department of Microbiology, Immunology and Transplantation, KU Leuven, 3000 Leuven, Belgium; 2 Laboratory of Hepatology, CHROMETA Department, KU Leuven, 3000 Leuven, Belgium; 3 Department of Abdominal Transplant Surgery, University Hospitals Leuven, 3000 Leuven, Belgium

**Keywords:** normothermic kidney perfusion, kidney preservation, inflammation, cytokines, kidney preservation, organ perfusion

## Abstract

The ideal composition of the perfusate for normothermic kidney perfusion is unknown, though the perfusate commonly used to perfuse human kidneys contains leukocyte-depleted packed red blood cells (RBC), as this is believed to prevent excessive inflammation. We performed a systematic search identifying 19 articles reporting on cytokine levels during normothermic pig or human kidney perfusion. Cytokine levels varied widely across the reported studies. No direct comparisons of perfusate cytokines during perfusion with RBC or whole blood were performed, and no data on how these levels are influenced by ischemia are available. Therefore, we compared perfusate IL-6, IL-1β, TNF-α, TGF-β, IL-10, IL-8, and CCL2 levels during 4 h normothermic pig kidney perfusion with a whole blood- or RBC-based perfusate. Kidneys were exposed to either 1 h of warm or 22 h of cold ischemia. We found no evidence of different perfusate cytokine or gene expression levels in whole blood or RBC perfusions. There was no clear evidence to suggest that cytokine concentrations differ between ischemically injured kidneys and controls. In conclusion, pro-inflammatory and anti-inflammatory cytokines and chemokines are detectable in the perfusate and urine of kidneys undergoing normothermic perfusion. It is unclear how cytokine levels change in different ischemic conditions and whether the use of a leukocyte filter plays a role.

## 1. Introduction

Normothermic kidney perfusion is being developed as a strategy to preserve and resuscitate donor kidneys destined for transplantation [1,2]. In contrast to static cold storage on ice, normothermic perfusion provides an oxygenated perfusate to the kidney in near-physiologic conditions [3]. To support renal metabolism during perfusion, so that cellular processes can continue ex situ, the perfusion solution needs to contain an oxygen carrier [3]. The ideal composition of the perfusion solution is unknown, though currently the perfusate commonly used to perfuse human kidneys contains leukocyte-depleted, packed red blood cells (RBC) [4]. A RBC-based solution is thought to be superior to the use of whole blood (WB), as the former might reduce the inflammatory response during perfusion. Indeed, during WB perfusion, all components of the inflammatory cascade are present, while this is not the case in RBC perfusion. However, tissue-resident immune cells are abundant in the kidney [5]. There is diapedesis of T cells, B cells, natural killer cells, and monocytes from the kidney into the circuit during normothermic kidney perfusion [6]. These cells are likely to mount a response during perfusion. This is evidenced by the detection of cytokines during perfusion and the fact that removing inflammatory cytokines from the perfusate, by a hemoadsorber, has been shown to attenuate inflammatory gene expression [7].

Here, we report on a systematic literature search to identify what is known about cytokine release during normothermic kidney perfusion. We found that no direct comparisons of perfusate inflammatory markers during normothermic kidney perfusion with RBC or WB over time have been reported. Furthermore, we found little information on how ischemia influences this cytokine release. We therefore present the results of a pilot study comparing cytokine levels during normothermic perfusion of pig kidneys with a WB- or RBC-based perfusate in which kidneys were exposed to either warm or cold ischemia.

## 2. Materials and Methods

### 2.1. Scoping Review

#### 2.1.1. Systematic Literature Search Strategy

This review was conducted using the Preferred Reporting Items for Systematic Reviews and Meta-Analyses (PRISMA) guidelines. The protocol was prospectively registered in the Open Science Framework [8]. PubMed, Embase, and Web of Science Core Collection were searched from inception. The following concepts: “cytokines”, “kidney”, and “perfusion” were developed. The complete search strategy can be found in Table S1.

#### 2.1.2. Study Selection, Eligibility Criteria, and Study Outcomes

The results of the search were imported into Endnote (Version X20, Clearview Analytics, Philadelphia, PA, USA), where they were screened for eligibility by two independent reviewers according to predefined inclusion and exclusion criteria (Table S2). Any disagreements were solved between them. Duplicates were removed using the “Find duplicates” tool in Endnote, and screening was performed in Rayyan [9]. Articles written in a language other than English, Dutch, or French; articles with no full text available; review articles; letters; editorials; and conference abstracts were excluded. Only studies evaluating cytokines of isolated pig or human kidneys (both ex situ and in situ) during normothermic perfusion were included. Reference lists of included studies were also searched using the same inclusion and exclusion criteria (“snowballing”).

#### 2.1.3. Data Extraction

The full data extraction table is freely accessible and contains information on title, authors, year of publication, study type, experimental set-up, group characteristics, perfusion characteristics, timing and type of sample collection, analysis techniques, analysed cytokines, and key findings [10]. Cytokine concentrations in perfusate and urine at each time point were extracted from graphs using WebPlotDigitizer v.4.3 (Ankit Rohatgi, Pacifica, CA, USA) (Suppl Methods) and are freely available [10].

### 2.2. Experiments

This study makes use of biobanked perfusate samples collected during the conduct of normothermic pig kidney perfusion, performed according to the European guidelines [11] and approved by the Animal Ethics Committee of KU Leuven (P209/2017).

#### 2.2.1. Animal Experiments

Kidneys were retrieved from male, prepubescent pigs (TOPIGS-TN70, Tojapigs Escharm, Nijmegen, The Netherlands), weighing 35 to 45 kg (Supplemental Methods). After nephrectomy, the renal artery was cannulated, and the kidney was flushed with 250 mL of cold preservation solution (IGL-1 at 4 °C, Institut Georges Lopez, Lissieu, France). The infrarenal aorta was then punctured to obtain 500 mL of autologous blood for normothermic perfusion. Arterial blood was drained by gravity into a heparinised (3 mL, Heparine Leo^®^, Leo Pharma, Lier, Belgium) collection bag. Before mounting on the perfusion circuit, kidneys were rinsed with 200 mL of cold Ringer’s solution (4 °C).

#### 2.2.2. Experimental Groups

Two perfusion solutions were studied: autologous whole blood perfusion (WB) or perfusion with an autologous RBC-based perfusate. For each perfusion solution, three experimental groups were investigated: (a) control kidneys that were mounted on the normothermic perfusion circuit immediately; (b) cold ischemic kidneys (CI) were submerged in IGL-1 and stored on ice for 22 h; (c) warm ischemic kidneys (WI) underwent 60 min of in situ warm ischemia (ligation of renal artery and vein) before nephrectomy. Each group contained 3 kidneys, except RBC-CI, in which 4 kidneys were included.

To recover RBCs separately, WB was washed with NaCl 0.9% (Autolog autotransfusion system, Medtronic, Jette, Belgium). WB or RBCs were added to the perfusion circuit immediately (in Control and WI kidneys) or stored at 4 °C until use (in CI kidneys).

#### 2.2.3. Porcine Normothermic Kidney Perfusion Model

Kidneys underwent normothermic perfusion for 4 h on a custom-made circuit (Suppl Methods). Arterial pressure was maintained between 60 and 70 mmHg by manually adjusting perfusion flows.

The perfusion circuit was primed with Ringer’s solution and colloid albumin (Human Albumin 20%, CAF-DCF, Brussels, Belgium) (Table S3). The priming solution was supplemented with either WB or RBC. Urine was replaced by Ringer’s solution in a 1:1 fashion.

#### 2.2.4. Sample Collection

A sample line was connected to the arterial inflow cannula, and perfusate samples were collected in ethylenediaminetetraacetic acid (EDTA) tubes and centrifuged at 1000× *g* for 10 min at 4 °C, aliquoted (1 mL), snap frozen in liquid nitrogen, and stored at −80 °C until analysis. Cortical tissue was collected by a 14 G needle biopsy gun right before mounting the kidney on the circuit and at the end of perfusion. The tissue was snap frozen in liquid nitrogen and stored at −80 °C until analysis.

#### 2.2.5. Perfusate Analyses

To evaluate kidney function, a creatinine bolus was added to the perfusion circuit (Table S3) that was cleared from the perfusate by the kidney over time. Cellular injury was quantified by measuring aspartate aminotransferase (AST) and heart-fatty acid binding protein (h-FABP), a marker of distal tubular injury, in the perfusate at 2 and 4 h of perfusion [12]. Cytokine and chemokine concentrations were measured at 2 and 4 h of perfusion. Pro-inflammatory cytokines Interleukin-6 (IL-6), IL-1β, and Tumor Necrosis Factor-alpha (TNF-α) were determined, as well as anti-inflammatory cytokines IL-10 and Transforming Growth Factor-beta (TGF-β), and chemokines IL-8 and Monocyte Chemoattractant Protein (CCL2).

Perfusate creatine and AST levels were enzymatically determined by the central laboratory of the University Hospitals Leuven (creatininase peroxidase and IFCC method, respectively on COBAS 8000 Hitachi/Roche). Enzyme-linked immunosorbent assay (ELISA) was used to determine the concentration of all other parameters (Table S4). Creatinine, AST, and h-FABP were corrected for kidney weight. Cytokine and chemokine concentrations were corrected for total protein concentration in the sample. Protein concentrations were determined by the Bradford protein assay [13].

#### 2.2.6. Quantitative Real-Time Quantitative Polymerase Chain Reaction

RNA was extracted from kidney tissues using Trizol reagent and chloroform, followed by an additional purification step with the RNeasy mini kit (Qiagen, Hilden, Germany), according to the manufacturer’s instructions. The mRNA expression levels in kidney tissues were analyzed using quantitative real-time polymerase chain reaction (Q-RT-PCR) (Suppl Methods). Experiments were carried out in duplicates. Using the 2^−∆∆Ct^ method [14], the relative quantification in gene expression was determined. Data are expressed as the relative differences (fold change) between the baseline and 4 h samples after correction for actin expression.

#### 2.2.7. Statistical Analysis

Data are presented as individual values or summarized as mean ± standard deviation (SD). Cytokine concentrations below the lower limit of detection (LLOD) were imputed by the LLOD/2 and corrected for the dilution factor used in the assay, so h-FABP: 19.55 ng/mL, IL-6: 2250 pg/mL, IL-1β: 3.35 pg/mL, TNF-α: 185 pg/mL, IL-10: 3.0 pg/mL, TGF-β: 4.9 pg/mL, IL-8: 23 pg/mL, CCL2: 2800 pg/mL. Four of the seven cytokines had levels below the LLOD (33% to 100% of measurements), mostly at the 2 h timepoint (Table S5).

Clinical outcome values and injury markers were corrected for kidney weight (per 100 g of kidney). Cytokine and chemokine concentrations were corrected for total protein concentration (per 1 mg of protein) in the sample. Longitudinal data were summarized by the area under the curve (AUC). Differences between groups were tested with an unpaired *t*-test or a one-way ANOVA with post-hoc Tuckey, as appropriate. Within-group differences were tested with a paired *t*-test. A *p*-value of <0.05 in a two-sided test was considered significant. Statistical analyses were performed with SPSS (SPSS Inc., Chicago, IL, USA, version 20). Data were visualised using GraphPad Prism (GraphPad Software, San Diego, CA, USA, version 9).

## 3. Results

### 3.1. Results of the Systematic Literature Search

A systematic search of online databases, performed on 22 July 2022, identified 719 records, of which 18 papers met the inclusion and exclusion criteria. From the reference lists, another 528 potential papers were identified, of which one was included (Figure S1 shows the flow chart). In total, 19 articles were included in this review [6,7,15,16,17,18,19,20,21,22,23,24,25,26,27,28,29,30,31]. All reported on experimental studies (15 pig kidneys [6,15,16,17,18,19,20,21,22,23,24,25,26,27,30], four human kidneys assessed as not suitable for transplantation [7,28,29,31]), one study also included data from transplanted human kidneys [7] (Table 1). Cytokines were mostly investigated in the perfusate (n = 10 [6,7,20,22,24,26,27,28,30,31]), followed by urine (n = 6 [7,15,16,17,18,19,20,21,23,25,29]) and tissue (n = 5 [7,15,17,20,29]). The majority of kidneys had been exposed to warm ischemia (ranging from 5 min to 75 min in pig studies and up to 138 min in human kidneys; Table S6) followed by a variable period of cold ischemia (2 h to 24 h in pig studies and up to 47 h in human kidneys; Table S6), except in one study in which kidneys were not stored but immediately placed on the perfusion device [24].

#### 3.1.1. Perfusate Cytokines

Cytokine concentrations vary highly between studies, but most studies describe an increase in pro-inflammatory cytokines over time (Figure S2, Table 1). The observed variability across studies might be related to the varying study set-up (e.g., i.e., the length of warm and cold ischemia time, the composition of the perfusate (Table S6). Because of this variability in study design, we present cytokine changes over time per study in Figure S2 and did not aggregate data in a meta-analysis. The most frequently measured perfusate cytokines are IL-1β, IL-6, IL-10, and TNF-α, though IL-1α, IL-1ra, IL-2, IL-4, IL-8, IL-12, IL-18, CCL2, CCL4, and CXCL8 concentrations have also been determined.

#### 3.1.2. Urinary Cytokines

Urinary cytokine measurements were limited to a single time point per study (at 3 h or 4 h of perfusion). Hosgood et al. investigated the effect of different lengths of warm and cold ischemia time on urinary IL-6 and TNF-α concentrations at 3 h of perfusion [18]. Levels of both cytokines were significantly higher in kidneys stored for 18 h after 10 min and 25 min of warm ischemia compared with kidneys without warm ischemia. When visualising data from all studies, there seems to be some suggestion that urinary cytokine concentrations increase with increasing warm and cold ischemia time, though these data need to be interpreted carefully, as they originate from different studies (Figure S3).

#### 3.1.3. Gene Expression Changes

Data from human kidneys show upregulation of genes IL1B, IL6, IL8, and TNF during perfusion (Table 1).

### 3.2. Experimental Study

#### 3.2.1. Ischemically Injured Kidneys Behave Similarly during Normothermic Perfusion with Whole Blood Compared to Concentrated Red Blood Cells

“On-pump” kidney function, as assessed by the clearance of creatinine over the 4 h perfusion period, was distinctly and statistically different between the three ischemic conditions in both whole blood and RBC perfusions (Figure S4A,B). Ischemically injured kidneys also had lower oxygen consumption compared to controls in both whole blood and RBC perfusions (Figure S4A,B) and higher levels of injury markers AST and h-FABP, though these differences were not always statistically significant, likely because of sample size (Figure S4B). We found no clear evidence to suggest that either perfusate resulted in markedly improved kidney function or reduced injury (Figure S4C).

#### 3.2.2. Cytokines Changes during Perfusion with WB and RBC Perfusate

**Figure 1:**
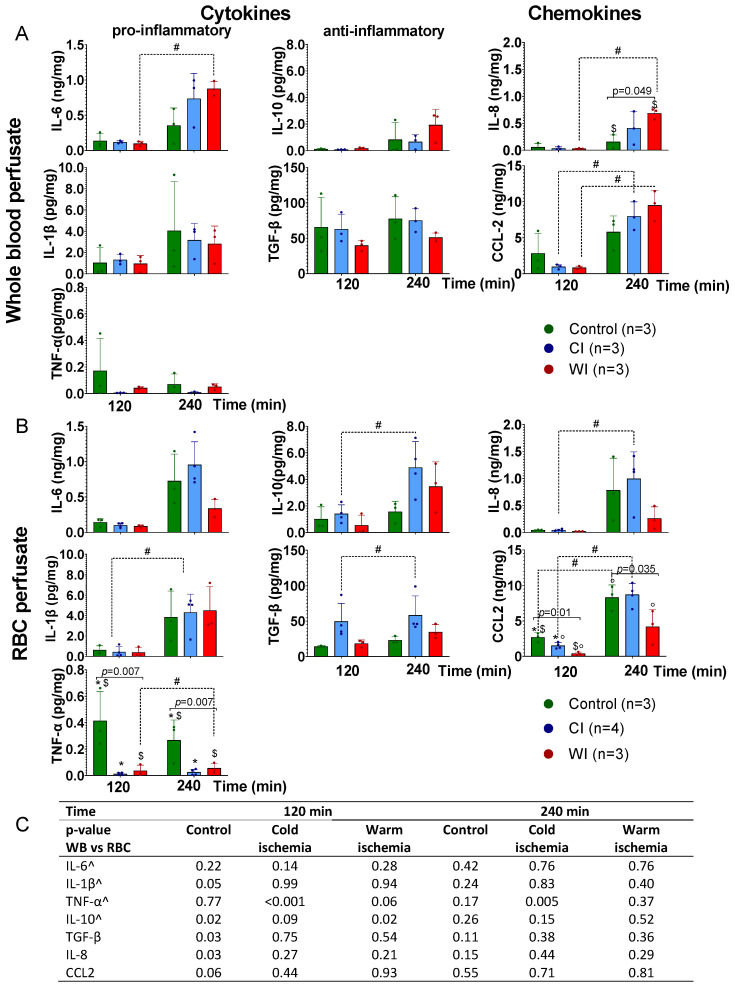
Perfusate cytokine concentrations during normothermic perfusion of pig kidneys exposed to different ischemic injury and perfused with either a whole blood or red blood cell based perfusate. (**A**) Perfusion with autologous whole blood. Pig kidneys were exposed to no important ischemic insults (control); 22 h of cold ischemia (CI) mimicking clinical kidney transplantation; or 60 min of warm ischemia (WI) mimicking anoxic/hypoxic acute kidney injury). (**B**) Perfusion with a concentrated autologous red blood cell perfusate (Table S1). (**C**) Perfusate cytokine concentrations were comparable between perfusions with whole blood and red blood cells, and the same was true for each experimental condition. Significant pairwise comparisons are denoted by a dotted line, in which # indicates a *p* < 0.05; significant comparisons between ischemic conditions are denoted by a full line, and exact *p*-values with significant posthoc comparisons indicated by * for control vs. CI, ° CI vs. WI, and $ control vs. WI. ^ denotes cytokines with a considerable number of measured concentrations below the lower limit of detection (Table S5). CI, cold ischemia; RBC, red blood cells; WB, whole blood; WI, warm ischemia.

Overall, perfusate cytokine levels were low, with a considerable number of measurements below the LLOD for IL-1β, IL-6, IL-10, and TNF-α (Table S5). In both WB and RBC perfusions, a numerical increase in concentration over time was observed for all cytokines across experimental groups, except for TNF-α, for which concentrations seemingly decreased in the control group. Only some of these changes over time were significant (Figure 1A,B). In both WB and RBC perfusions, cytokine concentrations at 2 h of perfusion were similar in all groups, except for TNF-α and CCL2, for which higher concentrations in the control were detected compared to ischemically injured kidneys (Figure 1A,B). There is no clear evidence to suggest that cytokine concentrations at 4 h of perfusion differ between ischemically injured kidneys and control kidneys (Figure 1A,B). Additionally, these data show little evidence to suggest that perfusate cytokine concentrations differ between WB and RBC perfusions (Figure 1C). We observed an increased gene expression of both pro-inflammatory (TNF-α and IL-8) and anti-inflammatory (IL-10 and TGF-β) genes at 4 h of perfusion compared to the start of perfusion, with some suggestion of significantly higher gene expression in CI kidneys (Figure S5). There was no significant difference in gene expression between WB and RBC perfusions.

## 4. Discussion

Our systematic literature search showed that pro-inflammatory and anti-inflammatory cytokines as well as chemokines are released in the perfusate and urine during normothermic kidney perfusion. This is the case for perfusions with WB but also for those making use of a RBC-based solution and even those making use of a leukocyte filter. Overall, perfusate cytokine concentrations varied widely between studies, with many being undetectable at the start of perfusion. This large variation in cytokine concentrations between the different studies might be related to different lengths of perfusion, ischemic conditions, composition of the perfusate, sample handling, etc. It might also be that donor type (i.e., DBD with its cytokine storm or DCD with a catecholamine storm) influences cytokine levels during normothermic perfusion. It is also known that cytokine concentrations are affected by sample medium, freeze–thaw cycles, and length of storage [32,33]. This also makes it difficult to compare findings across studies, though most studies describe an increase in pro-inflammatory cytokines over time, and this was confirmed in our own study. This increase was observed in both WB and RBC perfusions and all investigated ischemic conditions.

We found no evidence to suggest that perfusate cytokine or gene expression levels were different in WB or RBC perfusions. We also found no convincing evidence that a RBC-based perfusate improved kidney function during perfusion compared to WB. Our systematic search could not identify any other study that directly compared cytokine concentrations between WB- and RBC-based perfusates, nor whether the use of a leukocyte filter reduces the perfusate cytokine concentrations. One study showed an increased expression of the IL-1β active subunit in the tissue 6 h after WB perfusion that was partially reduced by the use of a leukocyte filter, although the IL-1β precursor increased regardless of the use of a leukocyte filter [15].

We found no clear indication that ischemic conditions influence cytokine concentrations, but this needs to be investigated further. We identified only one other report assessing the effect of warm and cold ischemia on cytokine concentrations, but in this study, only urinary cytokine levels were determined [18]. This study found higher levels of urinary IL-6 and TNF-α in the kidney with 10 and 25 min of warm ischemia before cold storage [18]. From the aggregated data extracted from the papers identified in the systematic search, we found no clear indication suggesting that cytokine concentrations increase with increasing cold ischemia time, though interpretation of the findings is hampered by the different study designs (Figure S2).

As with all systematic searches, it is possible that some relevant articles were not identified or that relevant studies were published after the search. We limited the possibility of missing relevant articles by setting up a broad search strategy with final searches conducted recently. Furthermore, references of included articles were searched to identify any articles that might have been missed in the systematic search.

Our pilot study has limitations, because the groups were small, and therefore, the data need to be interpreted with caution. Overall, perfusate cytokine levels were low in our study, with a considerable number below the LLOD at 2 h of perfusion. Nevertheless, measured levels were comparable to those reported by Hosgood et al., the only other study expressing cytokine levels corrected for total protein in the sample [22]. As perfusate compositions—and protein content in the perfusate—might influence cytokine concentrations, we standardized cytokine concentrations for total protein content.

In conclusion, pro-inflammatory and anti-inflammatory cytokines and chemokines are detectable in the perfusate and urine of kidneys undergoing normothermic perfusion. It is unclear whether and how cytokine levels change in different ischemic conditions and whether the use of a leukocyte filter plays a role.

## Figures and Tables

**Table 1:** Overview of study set-up, sampling times, and cytokines that were investigated, and findings of studies identified by the systematic search.

Reference	Species	Kidney Condition	Perfusate	Timepoints	Analysing Technique	Analysed Cytokines	Findings
**Perfusate studies**							
Stone 2016 [6]	Pig (n = 10)	WI + CI	RBC + filter	0 h, 1 h, 6 h	Luminex	IL-1α, IL-1β, IL-2, IL-4, IL-6, IL-10, IL-12, IL-18, TNF-α, CXCL-8	Cytokines are undetectable at 0 h with virtually none (Il-1α, IL-4) or increase (IL-1β, IL-2, IL-6, IL-10, IL-12, ILI-18, TNF-α, CXCL-8) at 6 h
Hosgood 2017 ^1^ [20]	Pig (n = 5)	CI	WB	0 h, 1 h, 3 h, 6 h	ELISA	IL-1α, IL-1β, IL-6, IL-8, IL-10, TNF-α	IL-1α mostly undetectable (one outlier), the other cytokines increase (some) over time.
Hosgood 2018 [22]	Pig (n = 5)	W + CII	WB	0 h, 3 h, 6 h	ELISA	IL-1β, IL-6, IL-8	Significant increase at 3 h (IL-1β, IL-6) and 6 h (all cytokines) compared to baseline.
Bleilevens 2019 ^2^ [24]	Pig (n = 5)	WI	WB	0 h, 0.5 h, 1 h, 1.5 h, 2 h, 5 h, 6 h	ELISA	IL-6, IL-10, TNF-α	Significant increase of IL-6, no change in IL-8, and TNF-α increases until 2 h after which there is a seeming decrease.
Pool 2020 [26]	Pig (n = 5)	WI + CI	RBC + filter	0 h, 1 h, 2 h, 3 h, 4 h, 5 h, 6 h, 7 h	Luminex	IL-6, IL-8, IL-10, IL-17, TNF-α, CCL2, CCL4, CXCL10	IL-6, IL-8 increase over time with no change in IL-10, TNF-α, CCL2, and CCL4. IL-17 and CXCL10 are undetectable (not all cytokines have been measured at all time points).
Ferdinand 2021 ^1^ [7]	Human (discards) (n = 5)	DBD (n = 1), DCD (n = 4)	RBC	4 h	ELISA	IL-1β, IL-6, IL-8, IL-10, TNF-α	Cytokines are detectable at 4 h.
Lohmann 2021 [27]	Pig (n = 7)	WI + CI	RBC	0 h, 1 h, 2 h, 4 h	ELISA	IL-6, IL-10	Increase over time.
Thomson 2021 [28]	Human (discards) (n = 5)	DBD (n = 3), DCD (n = 2)	RBC	0 h, 1 h, 2 h, 4 h, 7 h	ELISA	IL-1α, IL-1β, IL-2, IL-6, IL-8, IL-10, IL-17,TNF-α	All cytokines increase over time.
Mellati 2022 [30]	Pig (n = 5)	WI + CI	WB	0 h, 1.5 h, 3 h	Luminex	IL-1α, IL-1ra, IL-1β, IL-6, IL-8, IL-12	IL-1α, IL-1β, IL-12 decrease over time while IL-1ra, IL-6, and IL-8 increase over time.
Weissenbacher 2022 [31]	Human (discards) (n = 12)	DBD (n = 7), DCD (n = 5)	RBC	1 h, 6 h	Luminex	IL-1α, IL-1β, IL-2, IL-4, IL-6, IL-8, IL-10, IL-12p40, IL-12p70, TNF-α	Concentrations of IL-1α, IL-1β, IL-2, IL-4, IL-12p40, IL-12p70 are probably over the detection limit (their concentrations are all 10.000 pg/mL); IL-6, IL-8, TNF-α increase over time; IL-10 decreases over time.
**Urine studies**							
Hosgood 2011 [16]	Pig (n = 6)	WI + CI	WB	3 h	ELISA	IL-6	IL-6 is detectable in urine at 3 h of perfusion.
Hosgood 2012 [18]	Pig (n = 5)	CI	WB	3 h	ELISA	IL-6, TNF-α	All are detectable in the perfusate after 2 h or 18 h of cold storage.
	(n = 6)	WI + CI	WB	3 h	ELISA	IL-6, TNF-α	All are detectable in the perfusate after 2 h or 18 h of cold storage.
Hosgood 2013 [19]	Pig (n = 6)	WI + CI	WB	3 h	ELISA	IL-1β, IL-6, IL-8, TNF-α	All are detectable in urine at 3 h of perfusion.
Smith 2017 [21]	Pig (n = 6)	WI + CI	WB + filter	3 h	ELISA	IL-6, IL-8, IL-10	Cytokines are detectable in urine at 3 h of perfusion, with large standard deviation.
Bhattacharjee 2019 [23]	Pig (n = 5)	WI + CI	WB	4 h	ELISA	IL-6	IL-6 is detectable in urine at 4 h of perfusion.
Bhattacharjee 2020 [25]	Pig (n = 5)	WI + CI	WB	4 h	ELISA	IL-6	IL-6 is detectable in urine at 4 h of perfusion.
**Tissue studies**							
Yang 2010 [15]	Pig (n = 6)	WI + CI	WB	Pre-perfusion, 6 h	Western Blot	IL-1β	Significant increase of IL-1β precursor and IL-1β subunit at 6 h compared to pre-reperfusion.
Yang 2011 [17]	Pig (n = 3)	WI + CI	WB + filter	Pre cold storage, post cold storage, 2 h	Western Blot	IL-1β	Significant increase of IL-1β precursor and IL-1β subunit at 6 h compared to pre-reperfusion.
Hosgood 2017 ^1^ [20]	Pig (n = 5)	CI	WB	In situ, 6 h	qPCR	IL-1β, IL-6, IL-8	No information on change compared to baseline.
Ferdinand 2021 ^1^ [7]	Human (transplants) (n = 5)	DBD (n = 1), DCD (n = 4)	RBC	0 h, 2 h	RNA sequencing	IL1B, IL8, TNF	Upregulation of these genes at 2 h compared to baseline.
	Human (discards) (n = 5)	DBD (n = 1), DCD (n = 4)	RBC	0 h, 2 h, 4 h	RNA sequencing	IL6, TNF	Upregulation of these genes over time.
Hosgood 2022 [29]	Human (discards) (n = 15)	DBD (n = 10), DCD (n = 5)	RBC	0 h, 1 h	qPCR	IL6	Upregulation of IL6 gene compared to baseline.

^1^ Assessed cytokines in multiple sample types (e.g., perfusate and tissue); ^2^ the corresponding author confirmed that cytokines are expressed as pg/mL (and not ng/mL as shown in the graph of the article) “filter” denotes a leukocyte filter CI, cold ischemia; DBD, donation after brain death; DCD, donation after circulatory death; WB, whole blood; WI, warm ischemia.

## Data Availability

The datasets have been deposited in RDR, KU Leuven’s data repository, and are publicly available [10,37].

## Supplementary Materials

The following are available online at https://www.mdpi.com/article/10.3390/jcm11226618/s1, Supplementary methodology: Figure S1: Flow chart of the systematic search identifying published papers reporting on cytokine measurements during normothermic perfusion of pig or human kidneys, Figure S2: Visual representation of perfusate cytokine concentrations extracted from articles identified by the systematic search, Figure S3: Visual representation of urine cytokine concentrations extracted from articles identified by the systematic search, Figure S4: Clinical read-outs and injury markers (AST, h-FABP) during normothermically perfusion of pig kidneys, Figure S5: Gene expression changes of pro-inflammatory and anti-inflammatory genes in cortex of normothermically perfused pig kidneys, Table S1: Search string in databases Pubmed, Embase, and Web of Science, Table S2: Inclusion and exclusion criteria, Table S3: Composition of the perfusate used for normothermic isolated kidney perfusion, Table S4: References of the ELISA kits used in the pig study, Table S5: Percentage of measurements below the lower limit of detection for each study group, timepoint, and cytokine, Table S6: Kidney preservation and normothermic perfusion setup as extracted from articles identified by the systematic search. Refs [34,35,36] cited in Supplementary Materials file.
