# Supplementary material for: Differential Cytokine Levels during Normothermic Kidney Perfusion with Whole Blood- or Red Blood Cell-Based Perfusates—Results of a Scoping Review and Experimental Study
Source: J Clin Med. 2022 Nov 8;11(22):6618. doi: 10.3390/jcm11226618 (PMC9695901; doi:10.3390/jcm11226618)
Supplement: Supplementary file 1 [file jcm-11-06618-s001.zip › jcm-1984228-supplementary.pdf]

Supplementary materials to

Cytokine changes during normothermic kidney perfusion with whole blood or red blood cell based perfusates – results of a scoping review and experimental study

Authors: Julie De Beule [1], Delphine Keppens [1], Hannelie Korf [2], Ina Jochmans [1,3]

Affiliations:

1 Department of Microbiology, Immunology, and Transplantation, Transplantation Research Group, Lab of Abdominal Transplantation, KU Leuven, Leuven, Belgium
2 Hepatology, Department of Chronic Diseases and Metabolism, KU Leuven, Leuven, Belgium
3 Department of Abdominal Transplant Surgery, University Hospitals Leuven, Leuven, Belgium

Content

Supplemental Methods…… P2
Table S1: Search string in databases Pubmed, Embase, and Web of Science…… P3
Table S2: Inclusion and exclusion criteria…… P3
Table S3: Composition of the perfusate used for normothermic isolated kidney perfusion…….. P4
Table S4: References of the ELISA kits used in the pig study…… P4
Table S5: Percentage of measurements below the lower limit of detection for each study group, timepoint, and cytokine…… P5
Table S6: Kidney preservation and normothermic perfusion setup as extracted from articles identified by the systematic search…… P6
Fig S1: Flow chart of the systematic search identifying published papers reporting on cytokine measurements during normothermic perfusion of pig or human kidneys…… P7
Fig S2: Visual representation of perfusate cytokine concentrations extracted from articles identified by the systematic search…… P8
Fig S3: Visual representation of urine cytokine concentrations extracted from articles identified by the systematic search…… P11
Fig S4: Clinical read-outs and injury markers (AST, h-FABP) during normothermically perfusion of pig kidneys…… P12
Fig S5: Gene expression changes of pro-inflammatory and anti-inflammatory genes in cortex of normothermically perfused pig kidneys…… P13
Acknowledgements…… P13
References …… P14

Supplemental Methods

Scoping Review

Data extraction from graphs during the Scoping Review

Cytokine concentrations in perfusate and urine at each time point were extracted from graphs using WebPlotDigitizer v.4.3 (Ankit Rohatgi, CA, USA) and are freely available [1]. If means or SD were not reported, other statistical variables (e.g., median, range) were used to infer them (Supplemental Methods). The SD can be directly obtained from the standard error or from the confidence interval for the mean. In other situations, an assumption about the distribution of the scores is needed. More specifically, medians were considered as means assuming a symmetric distribution. To obtain the mean and the SD if only the median and the range was reported, an approach proposed by Hozo et al. has been adopted [2]. If only the IQR was available, a normal distribution was assumed such that the SD could be obtained as IQR/1.35.

Experiments

Animal Experiments

Pigs were held in a specific-pathogen-free animal facility and allowed to accustom to the surroundings for at least 2 days before the experiment. Pigs were fasted 12 hours before surgery with ad libitum access to water. Pigs were sedated with an intramuscular injection of Tiletamine/Zolazepam (8 mg/kg, Zoletil®, Virbac, Belgium) combined with Xylazine (2 mg/kg, Xylazine®, VMD pharma, Belgium), anaesthetised by inhalation of isoflurane (1% Isovet®, Piramal Critical Care B.V., Belgium) followed by orotracheal intubation. Inhalation anaesthesia with isoflurane was continued and fentanyl (8 µg/kg, Fentanyl®, Janssen Pharmaceutica, Belgium) was infused at a constant rate. A laparotomy was performed and the kidney's pedicles were dissected free.

Porcine normothermic kidney perfusion model

Kidneys underwent normothermic perfusion for 4 hours on a custom made circuit. The perfusion circuit contained a kidney reservoir, a roller pump (Stöckert, Germany), a membrane oxygenator (Affinity Pixie with Cortiva Bioactive Surface (heparin coated), Medtronic, Belgium) and a heat exchanger, all connected by non-heparin coated polyvinylchloride tubing (Intersept® Class VI measures 1/4x1/16, Medtronic, Belgium). The kidney was connected to the pump via its arterial cannula containing an arterial pressure line to monitor perfusion pressures. Perfusate flows were measured by a flow probe (SonoTT Ultrasonic Flowcomputer, em-tec MEDICAL, Germany) positioned on the arterial inflow tubing. The renal vein drained freely into the reservoir. The ureter was cannulated and drained into a urine collector. Perfusion temperature was set at 38°C (normothermia in pigs) and an airflow of 100 mL/min O_2 at an FiO_2 of 21% was administered via the oxygenator.

Quantitative Real-Time Polymerase Chain Reaction

Reverse transcription was performed at 37°C for 1 h with the M-MLV Reverse Transcriptase (Invitrogen, Thermofisher Scientific, Waltham, USA) along with PCR nucleotide mix (Promega, Madisson, USA) and RNaseOUT (Invitrogen, Thermofisher Scientific, Waltham, USA). Q-RT-PCR was performed with the PCR mastermix of Applied Biosystems (1.503.193, Foster City, CA, USA). The following reagents from TaqMan Gene Expression Assays (Applied biosystems, Foster City, CA, USA) were used for RT-PCR experiments: β-actin (Ss03376563_uH), TNF-α (Ss03391318_g1), IL-8 (Ss03392435_m1), IL-10 (Ss03382372_u1), and TGF-β (Ss03382325_u1). Thermal cycling conditions

were composed of cDNA initially denatured at 95°C for 60 s, and then amplified by PCR for 45 cycles (95°C for 5 s, 60°C for 30 s).

Supplementary Tables

Table S1: Search string in databases Pubmed, Embase, and Web of Science

Database	Search string
**PubMed**	("Cytokines"[Mesh:NoExp] OR "Chemokines"[Mesh] OR "Interleukins"[Mesh] OR "Tumor Necrosis Factors"[Mesh] OR "cytokin*"[tiab] OR "interleukin*"[tiab] OR "tumor necrosis factor*"[tiab]) AND ("Kidney"[Mesh] OR "Kidney Transplantation"[Mesh] OR "kidney*"[tiab] OR "renal"[tiab]) AND ("Perfusion"[Mesh:NoExp] OR "perfus*"[tiab]) AND ("Swine" [Mesh] OR "Pigs"[Mesh] OR “swine”[tiab] OR "pig”[tiab] OR “pigs”[tiab] OR “Humans”[Mesh] OR “human*”[tiab])
**Embase**	(‘Cytokines’/de OR ‘Chemokines’/exp OR ‘Interleukins’/exp OR ‘Tumor Necrosis Factors’/exp OR ‘cytokin*’:ti,ab,kw OR ‘interleukin*’:ti,ab,kw OR ‘tumor necrosis factor*’:ti,ab,kw) AND (‘Kidney’/exp OR ‘Kidney Transplantation’/exp OR ‘kidney*’:ti,ab,kw OR ‘renal’:ti,ab,kw) AND (‘Perfusion’/de OR ‘perfus*’:ti,ab,kw) AND (‘Swine’/exp OR ‘Pigs’/exp OR ‘swine’:ti,ab,kw OR ‘pig’:ti,ab,kw OR ‘pigs’:ti,ab,kw OR ‘Humans’/exp OR ‘human*’:ti,ab,kw)<br>Filter: Articles, Conference articles
**Web of Science**	("cytokin*" OR "interleukin*” OR "tumor necrosis factor*”) AND ("kidney*" OR "renal") AND ("perfus*") AND (“swine” OR "pig” OR “pigs” OR “human*”)

Table S2: Inclusion and exclusion criteria

**Inclusion criteria**	1. Language: research articles in English, Dutch or French<br>2. Research articles: original research articles, systematic reviews without any restrictions of publication date or specific journals<br>3. Content:<br>a) Study must be performed in following species: pigs or humans<br>b) Only studies examining kidneys will be included<br>c) Kidneys have to undergo normothermic (≥35°C) [3] perfusion while being isolated from the rest of the body (i.e. either ex situ or in situ but with clear description of surgical isolation from the rest of the body/vasculature)<br>d) The cytokines studied should be either interleukins, chemokines, or tumor necrosis factors (as described by the MeSH terms of PubMed)<br>e) These cytokines should be studied in the perfusate<br>4. Full text available (freely online or via KU Leuven library)
**Exclusion criteria**	1. Language: other than English, Dutch, French<br>2. Study type: all papers that are not original research articles or systematic review, e.g., review articles, letter to editor, conference abstracts, editorials, …<br>3. Content:<br>a) Studies not using mammal kidneys (e.g. cell cultures) or kidneys from mammals other than pig or human<br>b) Only organs other than kidneys were examined<br>c) Studies where perfusion of the kidney is not isolated from the body (e.g. normothermic regional perfusion)<br>d) Studies where perfusion was not hypothermic perfusion (< 35°C) [3]<br>e) Studies not reporting on concentrations of interleukins, chemokines, or tumor necrosis factors in the perfusate<br>4. No full text available

Table S3: Composition of the perfusate used for normothermic isolated kidney perfusion

	Whole blood based	Red blood cell based
**Priming solution**		
Autologous whole blood	230 mL	
Autologous concentrated red cells		150 mL
Ringer's solution [a]	292 mL	335 mL
**Additives in bolus**		
Albumin 20%	58 mL	93 mL
Creatinine	145 mg	145 mg
Heparin	1250 IU	1250 IU
Glucose	270 mg (2.58 mM)	540 mg (5 mM)
Glutamine	42 mg (0.5 mM)	84 mg (1 mM)
**Infusions during perfusion**		
Glucose [b]	80 mg/h	80 mg/h
Glutamine [b]	24 mg/h	24 mg/h
Heparin [c]	50 IU/h	50 IU/h
Epoprostenol [d]	8,3 µg/h	8,3 µg/h

[a] Ringer's solution is composed of NaCl (8,6 g/L), KCl (0,3 g/L), CaCl2 (0,33 g/L); [b] 480 mg glucose and 144 mg glutamine dissolved in 50 mL of Ringer's solution; [c] 2500 IU dissolved in 50 mL of Ringer's solution; [d] 0.5 mg Epoprostenol dissolved in 10 mL of glycine buffer, 1.5 mL of this solution was then diluted in 7.5 NaCl 0.9%.

Table S4: References of the ELISA kits used in the pig study

Parameter	ELISA kit	Manufacturer	Lower limit of detection
h-FABP	Rat H-FABP (HK414)	Hycult, Biotech, Uden, the Netherlands	391 pg/mL
IL-1β	Porcine IL-1beta/IL-1F2 (PLB00B)	R&D Systems, bio-techne, Minneapolis, USA	6.7 pg/mL
IL-6	Swine IL-6 (ESIL6)	Invitrogen, Thermofisher Scientific, Waltham, USA	45 pg/mL
IL-8	Porcine IL-8/CXCL8 (P8000)	R&D Systems, bio-techne, Minneapolis, USA	4.6 pg/mL
IL-10	Swine IL-10 (KSC0101)	Invitrogen, Thermofisher Scientific, Waltham, USA	3.0 pg/mL
CCL2	Porcine CCL2/MCP-1 (ES2RB),	Invitrogen, Thermofisher Scientific, Waltham, USA	28 pg/mL.
TNF-α	Porcine TNF-alpha (PTA00)	R&D Systems, bio-techne, Minneapolis, USA	3.7 pg/mL
TGF-β	Mouse/Rat/Porcine/Canine TGF-β1 (MB100B)	R&D Systems, bio-techne, Minneapolis, USA	4.6 pg/mL

Table S5: Percentage of measurements below the lower limit of detection for each study group, timepoint, and cytokine

		WB			RBC		
**Cytokine**	**Timepoint**	**Control**	**CI**	**WI**	**Control**	**CI**	**WI**
IL-6	2h	33	0	33	0	25	33
IL-6	4h	0	0	0	0	0	0
IL-1β	2h	0	0	33	0	50	33
IL-1β	4h	0	0	0	0	0	0
TNF-α	2h	33	66	0	0	50	33
TNF-α	4h	33	66	0	0	50	0
IL-10	2h	66	100	33	0	0	33
IL-10	4h	33	33	0	0	0	0
TGF-β	2h	0	0	0	0	0	0
TGF-β	4h	0	0	0	0	0	0
IL-8	2h	0	0	0	0	0	0
IL-8	4h	0	0	0	0	0	0
CCL2	2h	0	0	0	0	0	0
CCL2	4h	0	0	0	0	0	0

Table S6: Kidney preservation and normothermic perfusion setup as extracted from articles identified by the systematic search

Reference	Species	WI (min)	CI (h)	Preservation method	Perfusion temperature (°C)	Pressure target (mmHg)	O_2 conc (%) / flow (l/min)	CO_2 conc (%) / flow (l/min)	Urine recirculation
Yang 2010 [4]	Pig	6 to 7	2	SCS	n.a.	55	n.a./n.a.	n.a./n.a.	No
Yang 2011 [5]	Pig	10	16	SCS	38	75	95/n.a.	5/n.a.	No
Hosgood 2011 [6]	Pig	10	18	SCS	38 to 39	85	n.a./n.a.	n.a./n.a.	No
Hosgood 2012 [7]	Pig	0, 10, 25	2, 18	SCS	38 to 39	n.a.	n.a./n.a.	n.a./n.a.	No
Hosgood 2013 [8]	Pig	10	24	SCS	38	85	95/0.5	5/0.5	No
Stone 2016 [9]	Pig	n.a.	2	SCS	38	75	95/0.5	5/0.5	No
Hosgood 2017 [10]	Pig	0	22	SCS	37,4	85	95/0.1	5/0.1	No
Smith 2017 [11]	Pig	15	17	SCS	38	n.a.	95/n.a.	5/n.a.	No
Hosgood 2018 [12]	Pig	15	22	SCS	37.4	85	25/0.1	5/0.1	No
Bleilevens 2019 [13]	Pig	Yes	0	None	38	75	n.a./n.a.	n.a./n.a.	No
Bhattacharjee 2019 [14]	Pig	30	4	SCS	37	70	40/n.a.	0/0	No
Bhattacharjee 2020 [15]	Pig	30	4	SCS	37	70	40/n.a.	0/0	No
Pool 2020 [16]	Pig	20	2 to 3	HMP	37	110/70	95/0.5	5/0.5	No
Ferdinand 2021 [17]	Human (transplants)	0 to 138	10 to 25.7	SCS	35.5 to 36.5	85	95/0.1	5/0.1	No
	Human (discards)	0 to 41	10 to 30.1	SCS	35.5 to 36.5	85	95/0.1	5/0.1	No
Lohmann 2021 [18]	Pig	75	14	HMP + O_2	37	70	95/0.5	5/0.5	No
Thomson 2021 [19]	Human (discards)	n.a.	13.1 to 36.3	SCS	36,5	75	n.a./n.a.	n.a./n.a.	No
Hosgood 2022 [20]	Human (discards)	9 to 18	8.9 to 34.2	SCS	35 to 37	75 to 85	n.a.	n.a.	No
Mellati 2022 [21]	Pig	at least 5	7	SCS	37	70	95/0.5	5/0.5	No
Weissenbacher 2022 [22]	Human (discards)	0 to 15	15.2 to 47	SCS	37	70 to 100	n.a./n.a.	n.a./n.a.	Yes

CI, cold ischemia; HMP, hypothermic machine perfusion; n.a., not available; WI, warm ischemia

Supplementary Figures

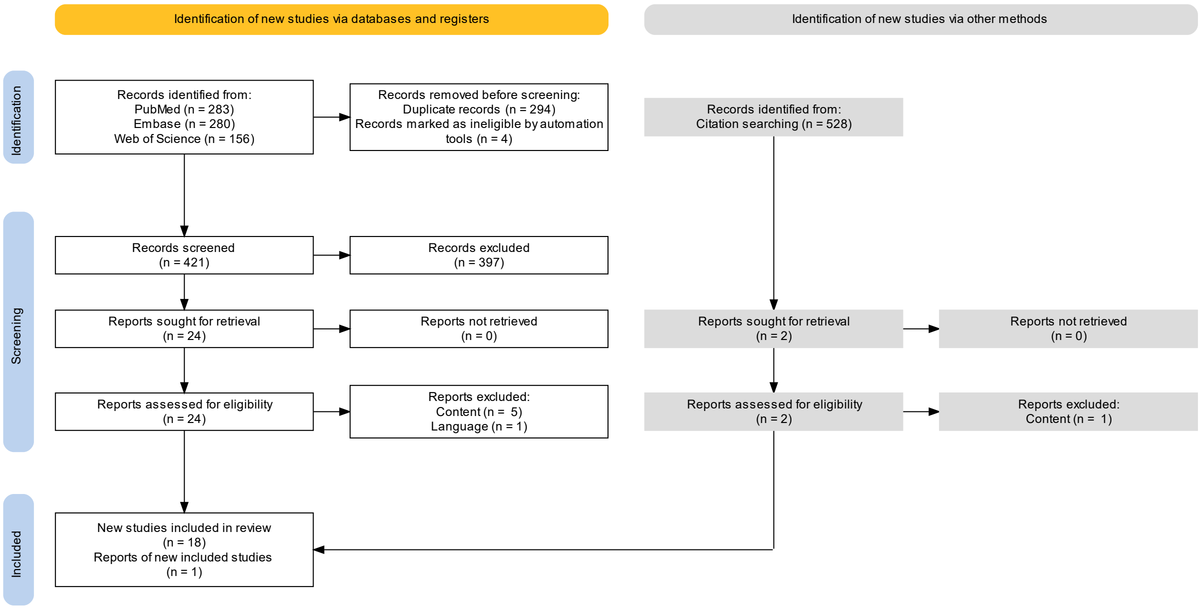

Fig S1: Flow chart of the systematic search identifying published papers reporting on cytokine measurements during normothermic perfusion of pig or human kidneys.
The Flowchart was created with the online tool PRISMA2020, created by Haddaway, N. R. et al. [23].

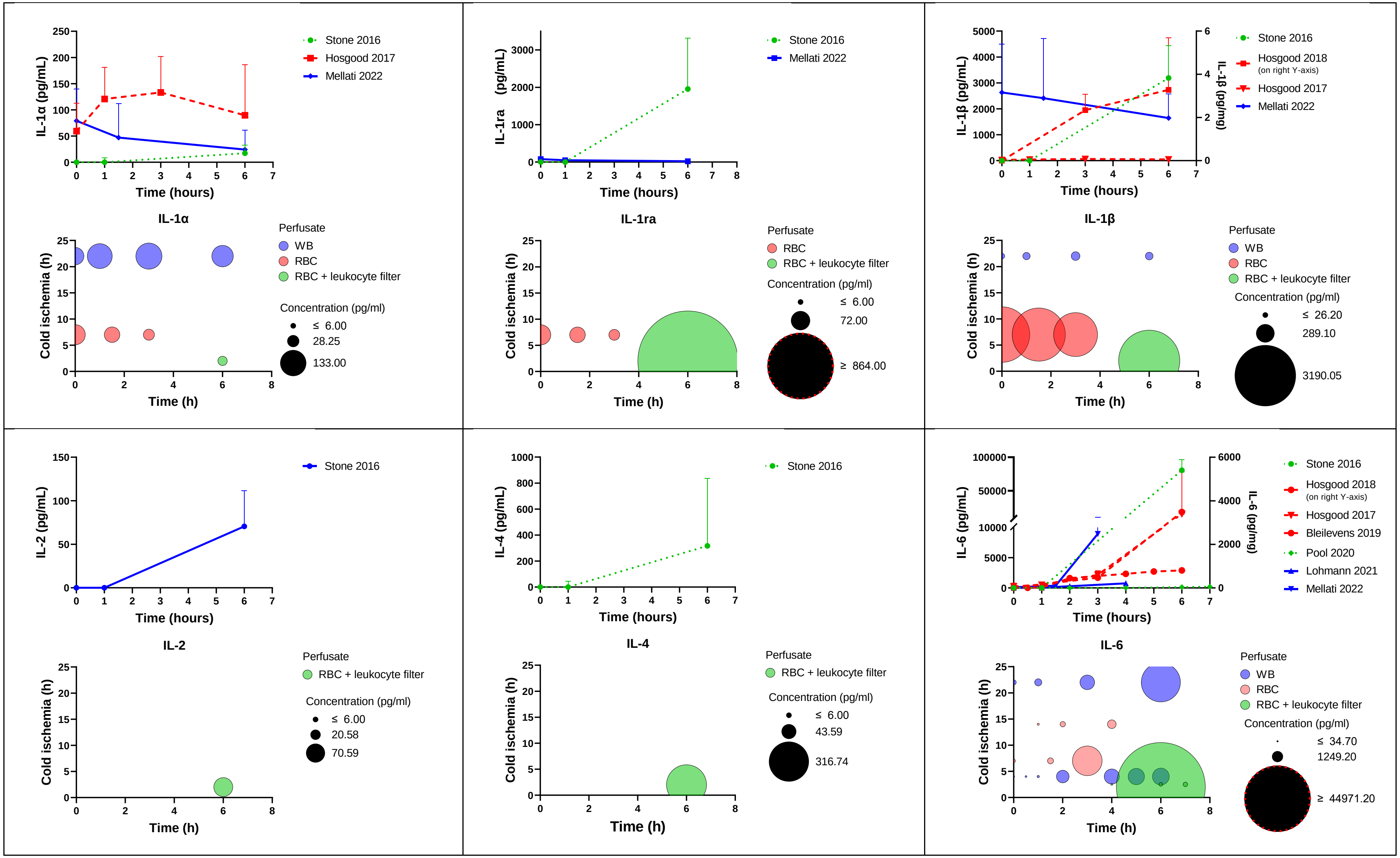

Stone 2016
Hosgood 2017
Mellati 2022
IL-1α (pg/mL)
Time (hours)
IL-1α
Perfusate
WB
RBC
RBC + leukocyte filter
Concentration (pg/ml)
≤ 6.00
28.25
133.00
Cold ischemia (h)
Time (h)
Stone 2016
Mellati 2022
IL-1ra (pg/mL)
Time (hours)
IL-1ra
Perfusate
RBC
RBC + leukocyte filter
Concentration (pg/ml)
≤ 6.00
72.00
≥ 864.00
Cold ischemia (h)
Time (h)
Stone 2016
Hosgood 2018
(on right Y-axis)
Hosgood 2017
Mellati 2022
IL-1β (pg/mL)
IL-1β (pg/mg)
Time (hours)
IL-1β
Perfusate
WB
RBC
RBC + leukocyte filter
Concentration (pg/ml)
≤ 26.20
289.10
3190.05
Cold ischemia (h)
Time (h)
Stone 2016
IL-2 (pg/mL)
Time (hours)
IL-2
Perfusate
RBC + leukocyte filter
Concentration (pg/ml)
≤ 6.00
20.58
70.59
Cold ischemia (h)
Time (h)
Stone 2016
IL-4 (pg/mL)
Time (hours)
IL-4
Perfusate
RBC + leukocyte filter
Concentration (pg/ml)
≤ 6.00
43.59
316.74
Cold ischemia (h)
Time (h)
Stone 2016
Hosgood 2018
(on right Y-axis)
Hosgood 2017
Bleilevens 2019
Pool 2020
Lohmann 2021
Mellati 2022
IL-6 (pg/mL)
IL-6 (pg/mg)
Time (hours)
IL-6
Perfusate
WB
RBC
RBC + leukocyte filter
Concentration (pg/ml)
≤ 34.70
1249.20
≥ 44971.20
Cold ischemia (h)
Time (h)

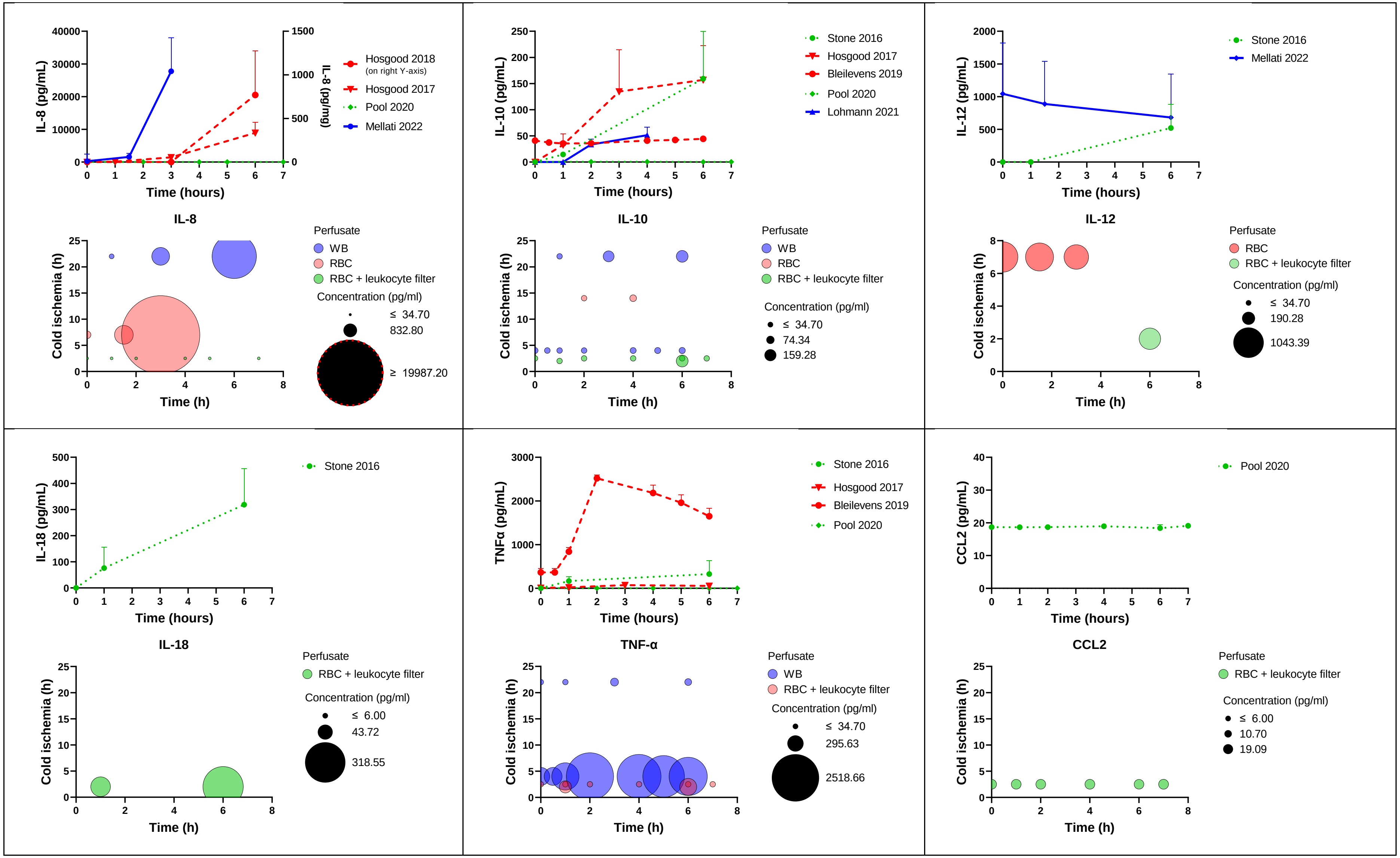

IL-8 (pg/mL)
IL-8 (pg/mg)
Time (hours)
Hosgood 2018
(on right Y-axis)
Hosgood 2017
Pool 2020
Mellati 2022
IL-8
Cold ischemia (h)
Time (h)
Perfusate
WB
RBC
RBC + leukocyte filter
Concentration (pg/ml)
≤ 34.70
832.80
≥ 19987.20
IL-10 (pg/mL)
Stone 2016
Bleilevens 2019
Lohmann 2021
IL-10
159.28
74.34
IL-12 (pg/mL)
IL-12
190.28
1043.39
IL-18 (pg/mL)
IL-18
≤ 6.00
43.72
318.55
TNFα (pg/mL)
TNF-α
295.63
2518.66
CCL2 (pg/mL)
CCL2
10.70
19.09

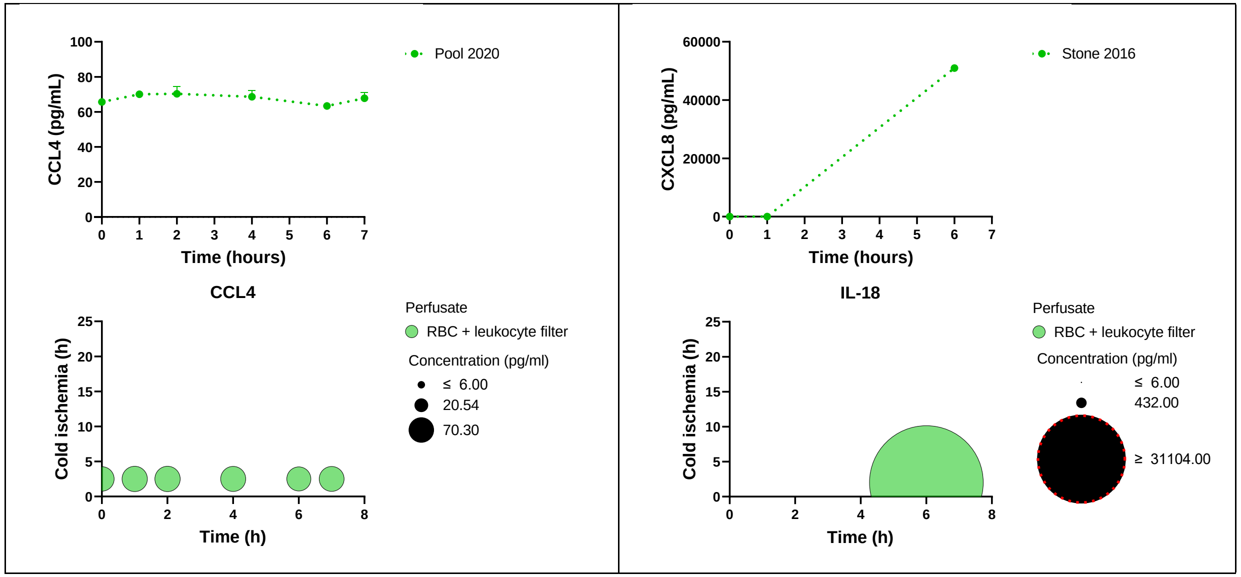

Fig S2: Visual representation of perfusate cytokine concentrations extracted from articles identified by the systematic search.
Each panel contains information on one cytokine. Within a panel, the upper graph represents mean concentrations (with standard deviation) over time for each study from which data could be extracted. Studies using a whole blood perfusate are represented with blue lines, those with a red blood cell based perfusate without a leukocyte filter in red with dashed lines, and those with a red blood cell based perfusate with a leukocyte filter in green with dotted lines. Within a panel, the lower graph is a bubble plot showing perfusion time in the x-axis and cold ischemia time in the y-axis. Each bubble represents a cytokine measurement, its area is proportional the concentration, the colour indicates the type of perfusion solution (blue for whole blood; red for red blood cell based, and green for red blood cell based with the use of a leukocyte filter, and the horizontal and vertical positions relate to perfusion time when the measurement took place and the cold ischemia time the kidney was exposed to, respectively. The studies contributing data to the bubble plot are those of which data is plotted in the upper graph of the panel.
RBC, red blood cell; WB, whole blood

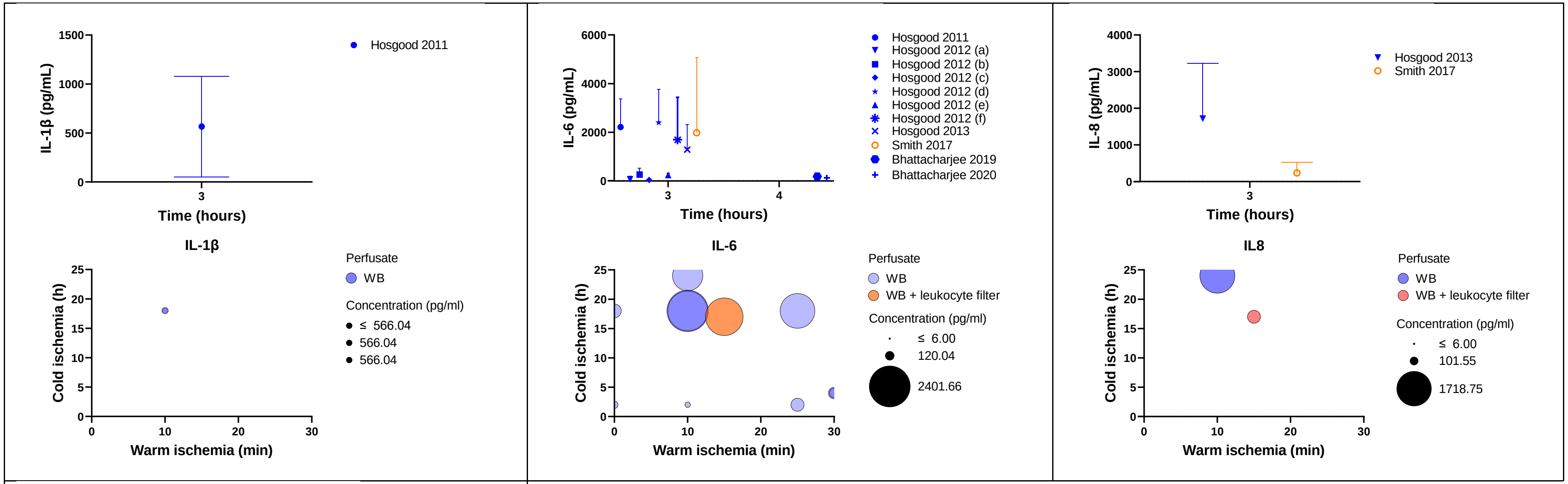

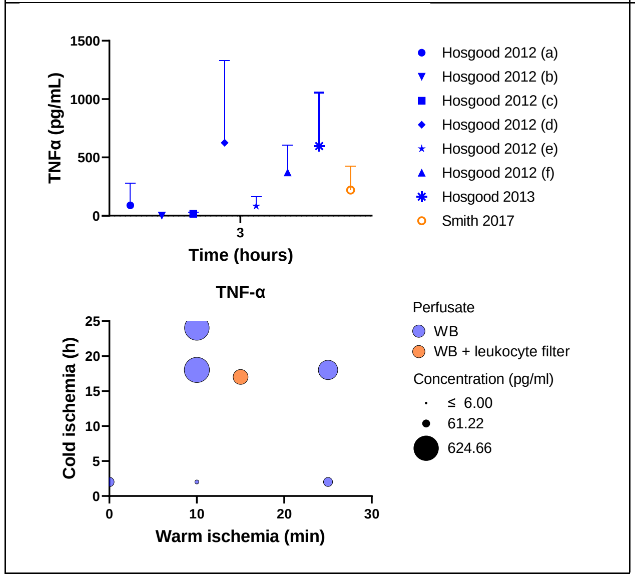

Fig S3: Visual representation of urine cytokine concentrations extracted from articles identified by the systematic search.

Each panel contains information on one cytokine. Within a panel, the upper graph represents mean concentrations (with standard deviation) over time for each study from which data could be extracted. Studies using a whole blood perfusate are represented in blue lines, those with a whole blood perfusate with a leukocyte filter in orange. Within a panel, the lower graph is a bubble plot showing warm ischemia time in the x-axis and cold ischemia time in the y-axis. Each bubble represents a cytokine measurement, its area is proportional the concentration, the colour indicates the type of perfusion solution (blue for whole blood; orange for whole blood with the use of a leukocyte filter, and the horizontal and vertical positions relate to warm ischemia time and cold ischemia time the kidney was exposed to, respectively. The studies contributing data to the bubble plot are those of which data is plotted in the upper graph of the panel.

Hosgood 2012 [7] has 6 groups: (a) 0 min WI + 2h CI; (b) 0 min WI + 18h CI; (c) 10 min WI + 2h CI; (d) 10 min WI + 18h CI; (e) 25 min WI + 2h CI; (f) 25 min WI + 18h CI

CI, cold ischemia; WB, whole blood; WI, warm ischemia

A

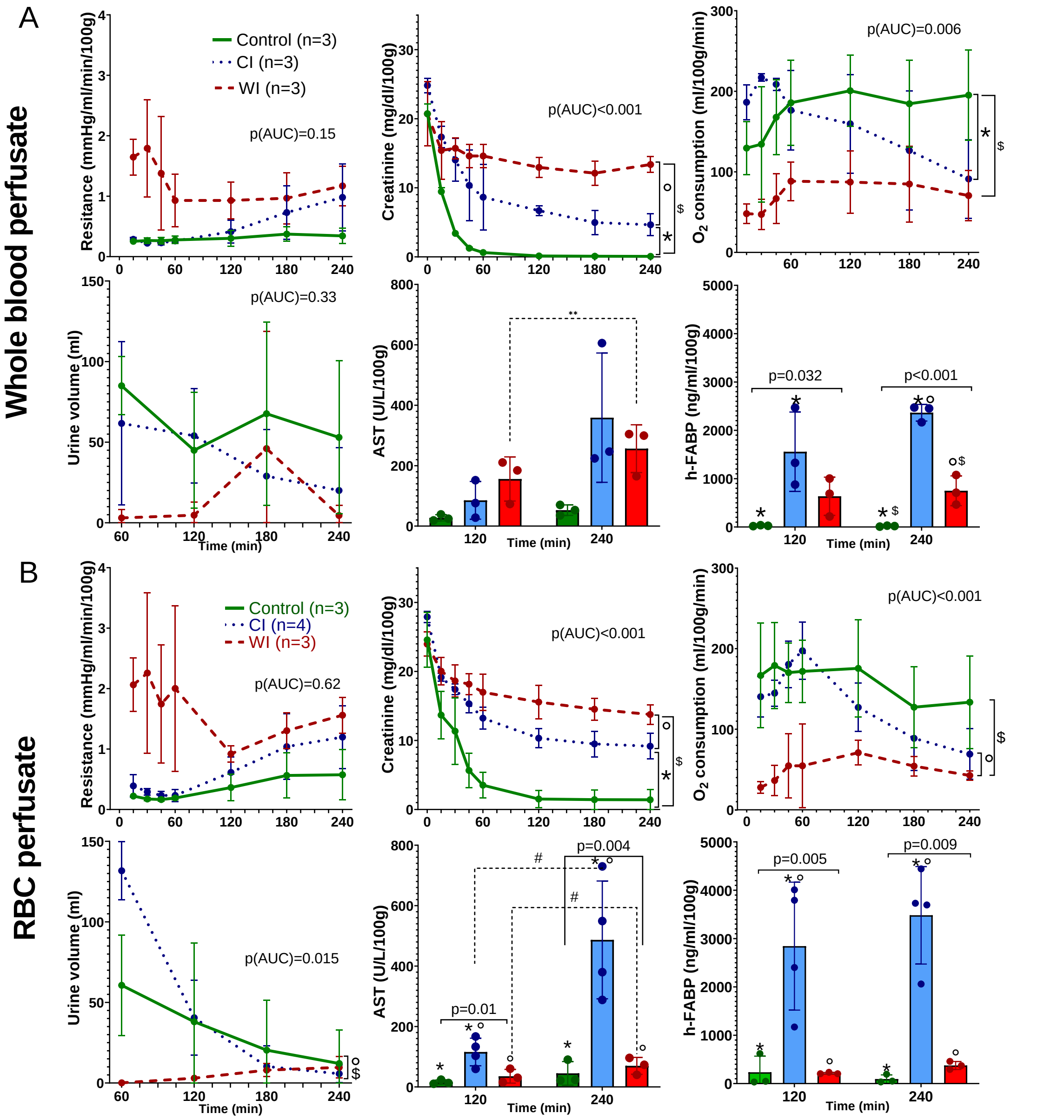

C

p-value WB vs RBC	Control	Cold ischemia	Warm ischemia
AUC Resistance over 4h	0.40	0.38	0.66
AUC Creatinine over 4h	0.03	0.07	1.00
AUC O_2 consumption over 4h	0.02	0.48	0.15
AUC Urine volume over 4h	0.45	0.11	0.14
AST at 2h	0.18	0.49	0.05
AST at 4h	0.64	0.45	0.02
h-FABP at 2h	0.41	0.74	0.18
h-FABP at 4h	0.40	0.91	0.24

Fig S4: Clinical read-outs and perfusate injury markers (AST, h-FABP) during normothermically perfusion of pig kidneys.

(A) Perfusion with autologous whole blood. Pig kidneys were exposed to no important ischemic insults (Control); 22h of cold ischemia (CI) mimicking clinical kidney transplantation; or 60 min of warm ischemia (WI) mimicking anoxic/hypoxic acute kidney injury); (B) Perfusion with a concentrated autologous red blood cell perfusate (Table S1); (C) Comparison between read-outs in kidneys perfused with whole blood or concentrated red blood cells. Significant pairwise comparisons are denoted by a dotted line where # indicates a p>0.05; significant comparisons between ischemic conditions are denoted by a full line and exact p-values with significant posthoc comparisons indicated by * for Control vs CI, ° CI vs WI, and $ Control vs WI.

AST, aspartate aminotransferase; AUC, area under the curve; CI, cold ischemia; h-FABP, heart-fatty acid binding protein; RBC, red blood cells; WB, whole blood; WI, warm ischemia

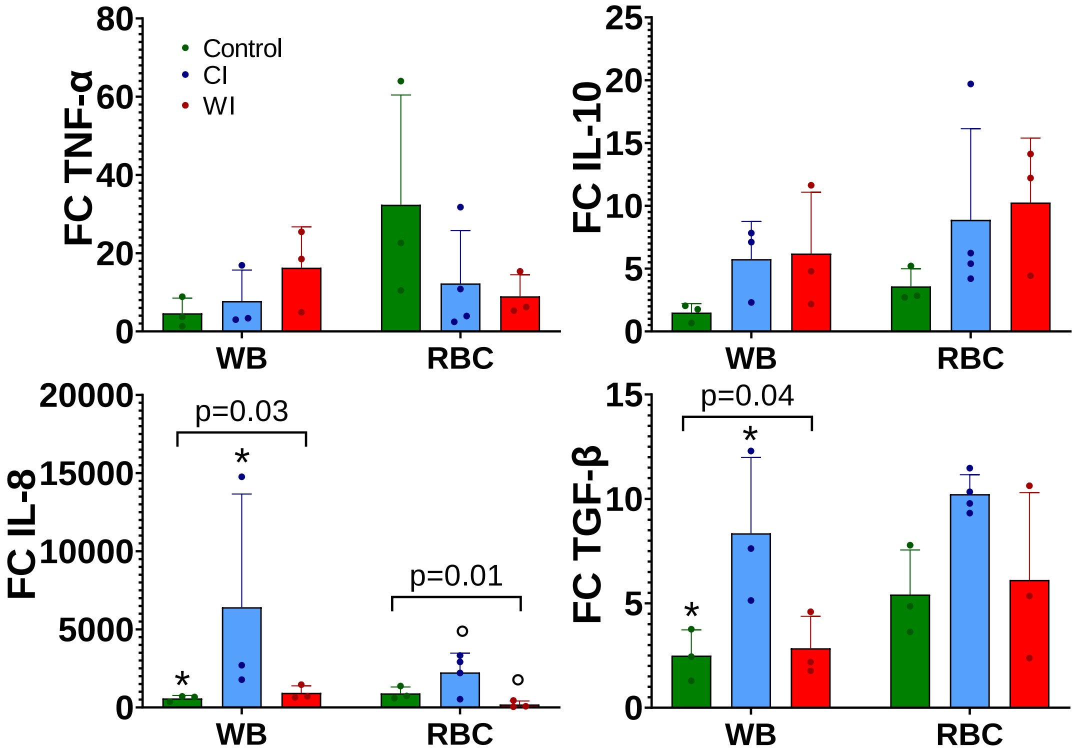

Fig S5: Gene expression changes of pro-inflammatory and anti-inflammatory genes in cortex of normothermically perfused pig kidneys.
Kidneys were perfused with autologous whole blood or a concentrated autologous red blood cell perfusate and exposed to no important ischemic insults (Control); 22h of cold ischemia (CI) mimicking clinical kidney transplantation; or 60 min of warm ischemia (WI) mimicking anoxic/hypoxic acute kidney injury). Data are expressed as the relative differences (fold change) between the baseline (right before the kidney is mounted on the device and perfusion is started) and 4h samples after correction for actin expression.
Significant pairwise comparisons are denoted by a dotted line where # indicates a p>0.05; significant comparisons between ischemic conditions are denoted by a full line and exact p-values with significant posthoc comparisons indicated by * for Control vs CI, ° CI vs WI, and $ Control vs WI.
CI, cold ischemia; RBC, red blood cells; WB, whole blood; WI, warm ischemia

Acknowledgements

We are grateful to the following student researchers who helped with sample collection during the perfusion experiments: Kevin Baar, Louise Bamelis, Sophie Beken, Noor Beneens, Marouane Benthami Kbibi, Laurens Bodard, Eva Borstlap, Olivier Bruynbroeck, Bertrand Cailliau, Maarten Cassiman, Evelyne Corthouts, Maxine de Corswarem, Thomas De Coster, Claire Defraigne, Stephanie Delcroix, Rutger Den abt, Jill Dewez, Maxim D'heedene, Lynn Goovaerts, Kyara Hamerlynck, Anthony Hanet, Lena Henneco, Fadi Hermez, Luxi Ji, Delphine Keppens, Lore Lambrechts, Lore Lemmens, Jozefien Lesage, Hanne Matheussen, Marianne Moens, Laila Mouqni, Stijn Noens, Yenthe Peeters, Nicola Peeters, Pieter Peeters, Laurence Ponsaerts, Bernard Roles, Jolyne Rutten, Astrid T'Jonck, Nam Cuong Trinh, Britt Valentyn, Laura Van Betsbrugge, Marco Garcia van Bijsterveld, Alyssa Van de Perre, Laure Van de Steen, Nicky van der Leeden, Robbe Van Dijck, Valerie Van Echelpoel, Inès Van Heesbeke, Sophie van Ockenburg, Xanthe Vanaken, Marlies Vandersnickt, Katho Vandewal, Anouck Vandoninck, Jef Vannetelbosch, Ruben Vanstiphout, Lisa Vanvynckt, Matthias Verholen, Annaël Vermeulen, and Maarten Warnants.

References

1. De Beule, J.; Jochmans, I. Replication Data for: Cytokine release during normothermic kidney perfusion – a scoping review. **2022**, doi:doi:10.48804/AELRQL, wil be accessible after acceptance of the paper.
2. Hozo, S.P.; Djulbegovic, B.; Hozo, I. Estimating the mean and variance from the median, range, and the size of a sample. *BMC Med Res Methodol* **2005**, *5*, 13, doi:10.1186/1471-2288-5-13.
3. Karangwa, S.A.; Dutkowski, P.; Fontes, P.; Friend, P.J.; Guarrera, J.V.; Markmann, J.F.; Mergental, H.; Minor, T.; Quintini, C.; Selzner, M.; et al. Machine Perfusion of Donor Livers for Transplantation: A Proposal for Standardized Nomenclature and Reporting Guidelines. *Am J Transplant* **2016**, *16*, 2932-2942, doi:10.1111/ajt.13843.
4. Yang, B.; Hosgood, S.A.; Harper, S.J.; Nicholson, M.L. Leucocyte depletion improves renal function in porcine kidney hemoreperfusion through reduction of myeloperoxidase+ cells, caspase-3, IL-1β, and tubular apoptosis. *J Surg Res* **2010**, *164*, e315-324, doi:10.1016/j.jss.2010.07.044.
5. Yang, B.; Hosgood, S.A.; Bagul, A.; Waller, H.L.; Nicholson, M.L. Erythropoietin regulates apoptosis, inflammation and tissue remodelling via caspase-3 and IL-1β in isolated hemoperfused kidneys. *Eur J Pharmacol* **2011**, *660*, 420-430, doi:10.1016/j.ejphar.2011.03.044.
6. Hosgood, S.A.; Mohamed, I.H.; Bagul, A.; Nicholson, M.L. Hypothermic machine perfusion after static cold storage does not improve the preservation condition in an experimental porcine kidney model. *Br J Surg* **2011**, *98*, 943-950, doi:10.1002/bjs.7481.
7. Hosgood, S.A.; Hunter, J.P.; Nicholson, M.L. Early urinary biomarkers of warm and cold ischemic injury in an experimental kidney model. *J. Surg. Res.* **2012**, *174*, e85-e90, doi:10.1016/j.jss.2011.10.024.
8. Hosgood, S.A.; Patel, M.; Nicholson, M.L. The conditioning effect of ex vivo normothermic perfusion in an experimental kidney model. *J Surg Res* **2013**, *182*, 153-160, doi:10.1016/j.jss.2012.08.001.
9. Stone, J.P.; Ball, A.L.; Critchley, W.R.; Major, T.; Edge, R.J.; Amin, K.; Clancy, M.; Fildes, J.E. Ex Vivo Normothermic Perfusion Induces Donor-Derived Leukocyte Mobilization and Removal Prior to Renal Transplantation. *Kidney Int. Rep.* **2016**, *1*, 230-239, doi:10.1016/j.ekir.2016.07.009.
10. Hosgood, S.A.; Moore, T.; Kleverlaan, T.; Adams, T.; Nicholson, M.L. Haemoadsorption reduces the inflammatory response and improves blood flow during ex vivo renal perfusion in an experimental model. *Journal of Translational Medicine* **2017**, *15*, doi:10.1186/s12967-017-1314-5.
11. Smith, S.F.; Adams, T.; Hosgood, S.A.; Nicholson, M.L. The administration of argon during ex vivo normothermic perfusion in an experimental model of kidney ischemia–reperfusion injury. *J. Surg. Res.* **2017**, *218*, 202-208, doi:10.1016/j.jss.2017.05.041.
12. Hosgood, S.A.; Moore, T.; Qurashi, M.; Adams, T.; Nicholson, M.L. Hydrogen Gas Does Not Ameliorate Renal Ischemia Reperfusion Injury in a Preclinical Model. *Artif. Organs* **2018**, *42*, 723-727, doi:10.1111/aor.13118.
13. Bleilevens, C.; Doorschodt, B.M.; Fechter, T.; Grzanna, T.; Theißen, A.; Liehn, E.A.; Breuer, T.; Tolba, R.H.; Rossaint, R.; Stoppe, C.; et al. Influence of vitamin C on antioxidant capacity of in vitro perfused porcine kidneys. *Nutrients* **2019**, *11*, doi:10.3390/nu11081774.
14. Bhattacharjee, R.N.; Ruthirakanthan, A.; Sun, Q.Z.; Richard-Mohamed, M.; Luke, S.; Jiang, L.; Aquil, S.; Sharma, H.; Tun-Abraham, M.E.; Alharbi, B.; et al. Subnormothermic Oxygenated Perfusion Optimally Preserves Donor Kidneys Ex Vivo. *Kidney Int. Rep.* **2019**, *4*, 1323-1333, doi:10.1016/j.ekir.2019.05.013.
15. Bhattacharjee, R.N.; Patel, S.V.B.; Sun, Q.Z.; Jiang, L.; Richard-Mohamed, M.; Ruthirakanthan, A.; Aquil, S.; Al-Ogaili, R.; Juriasingani, S.; Sener, A.; et al. Renal Protection Against Ischemia

Reperfusion Injury: Hemoglobin-based Oxygen Carrier-201 Versus Blood as an Oxygen Carrier in Ex Vivo Subnormothermic Machine Perfusion. *Transplantation* **2020**, *104*, 482-489, doi:10.1097/tp.0000000000002967.
16. Pool, M.B.F.; Vos, J.; Eijken, M.; van Pel, M.; Reinders, M.E.J.; Ploeg, R.J.; Hoogduijn, M.J.; Jespersen, B.; Leuvenink, H.G.D.; Moers, C. Treating Ischemically Damaged Porcine Kidneys with Human Bone Marrow- and Adipose Tissue-Derived Mesenchymal Stromal Cells During Ex Vivo Normothermic Machine Perfusion. *Stem Cells Dev.* **2020**, *29*, 1320-1330, doi:10.1089/scd.2020.0024.
17. Ferdinand, J.R.; Hosgood, S.A.; Moore, T.; Ferro, A.; Ward, C.J.; Castro-Dopico, T.; Nicholson, M.L.; Clatworthy, M.R. Cytokine absorption during human kidney perfusion reduces delayed graft function-associated inflammatory gene signature. *Am. J. Transplant.* **2021**, *21*, 2188-2199, doi:10.1111/ajt.16371.
18. Lohmann, S.; Pool, M.B.F.; Rozenberg, K.M.; Keller, A.K.; Moers, C.; Møldrup, U.; Møller, B.K.; Lignell, S.J.M.; Krag, S.; Sierra-Parraga, J.M.; et al. Mesenchymal stromal cell treatment of donor kidneys during ex vivo normothermic machine perfusion: A porcine renal autotransplantation study. *Am. J. Transplant.* **2021**, *21*, 2348-2359, doi:10.1111/ajt.16473.
19. Thompson, E.R.; Bates, L.; Ibrahim, I.K.; Sewpaul, A.; Stenberg, B.; McNeill, A.; Figueiredo, R.; Girdlestone, T.; Wilkins, G.C.; Wang, L.; et al. Novel delivery of cellular therapy to reduce ischemia reperfusion injury in kidney transplantation. *Am. J. Transplant.* **2021**, *21*, 1402-1414, doi:10.1111/ajt.16100.
20. Hosgood, S.A.; Elliott, T.R.; Jordan, N.P.; Nicholson, M.L. The Effects of Free Heme on Functional and Molecular Changes During Ex Vivo Normothermic Machine Perfusion of Human Kidneys. *Front. Immunol.* **2022**, *13*, 11, doi:10.3389/fimmu.2022.849742.
21. Mellati, A.; Lo Faro, L.; Dumbill, R.; Meertens, P.; Rozenberg, K.; Shaheed, S.; Snashall, C.; McGivern, H.; Ploeg, R.; Hunter, J. Kidney Normothermic Machine Perfusion Can Be Used as a Preservation Technique and a Model of Reperfusion to Deliver Novel Therapies and Assess Inflammation and Immune Activation. *Front. Immunol.* **2022**, *13*, 10, doi:10.3389/fimmu.2022.850271.
22. Weissenbacher, A.; Stone, J.P.; Lo Faro, M.L.; Hunter, J.P.; Ploeg, R.J.; Coussios, C.C.; Fildes, J.E.; Friend, P.J. Hemodynamics and Metabolic Parameters in Normothermic Kidney Preservation Are Linked With Donor Factors, Perfusate Cells, and Cytokines. *Front. Med.* **2022**, *8*, 14, doi:10.3389/fmed.2021.801098.
23. Haddaway, N.R.; Page, M.J.; Pritchard, C.C.; McGuinness, L.A. PRISMA2020: An R package and Shiny app for producing PRISMA 2020-compliant flow diagrams, with interactivity for optimised digital transparency and Open Synthesis. *Campbell Systematic Reviews* **2022**, *18*, e1230, doi:https://doi.org/10.1002/cl2.1230.
